# Validity of ultrasonography to assess hepatic steatosis compared to magnetic resonance spectroscopy as a criterion method in older adults

**DOI:** 10.1371/journal.pone.0207923

**Published:** 2018-11-26

**Authors:** Emanuella De Lucia Rolfe, Soren Brage, Alison Sleigh, Francis Finucane, Simon J. Griffin, Nick J. Wareham, Ken K. Ong, Nita G. Forouhi

**Affiliations:** 1 Medical Research Council Epidemiology Unit, University of Cambridge School of Clinical Medicine, Institute of Metabolic Science, Cambridge Biomedical Campus, Cambridge, United Kingdom; 2 Wolfson Brain Imaging Centre, University of Cambridge School of Clinical Medicine, and NIHR/Wellcome Trust Cambridge Clinical Research Facility, Cambridge University Hospitals NHS Foundation Trust, Cambridge, United Kingdom; 3 HRB Clinical Research Facility, National University of Ireland Galway, Galway, Ireland; 4 Department of Public Health and Primary Care, Institute of Public Health, University of Cambridge, Cambridge, United Kingdom; 5 Department of Paediatrics, University of Cambridge, Cambridge, United Kingdom; Universita degli Studi di Pisa, ITALY

## Abstract

**Background:**

The rising prevalence of obesity has made hepatic steatosis an increasingly common issue. Ultrasound is generally used in clinical practice to assess steatosis, but its accuracy has been inconsistent across studies. We aimed to determine the validity of ultrasound to diagnose hepatic steatosis when compared to the criterion method proton magnetic resonance spectroscopy (MRS) in older individuals.

**Methods:**

A total of 72 healthy white European individuals (n = 42 men; n = 30 women aged 67–76 years) participating in the Hertfordshire Birth Cohort Physical Activity trial had hepatic steatosis assessed by ultrasound and MRS. The ultrasound scans were graded as normal, mild, moderate and severe steatosis, while hepatic fat content above 5.5% by MRS was used as a cut-off for steatosis.

**Results:**

18 participants (25%) had a level of hepatic fat measured by MRS consistent with diagnosis of steatosis. The sensitivity and specificity of ultrasound in diagnosing hepatic steatosis (mild/moderate/severe vs normal) were 96% (95% CI: 87–99.6%) and 94% (95% CI: 73–100%) respectively, although overlap in MRS hepatic fat content was observed between the ultrasound categories.

**Conclusions:**

Ultrasound is a valid method for detecting the presence or absence of hepatic steatosis in older adults and can be used as an alternative tool in both clinical investigations and epidemiological studies, when other imaging techniques are not feasible.

## Introduction

Hepatic steatosis or fatty liver disease (FLD) is a condition characterised by triglyceride accumulation within the cytoplasm of hepatocytes [1]. Steatosis can progress to steatohepatitis (severe fatty liver accompanied by inflammation, with or without the presence of fibrosis) and cirrhosis (presence of hepatic fibrosis), and it has also been associated with an increased risk of hepatocarcinoma and cardiovascular disease [2–4].

Hepatic steatosis is related to excessive alcohol consumption (alcohol-related fatty liver disease) and obesity (non-alcoholic fatty liver disease–NAFLD). These two conditions are distinguished on the basis of alcohol intake with a threshold of <20 g alcohol per day in women and <30 g in men typically used to make a diagnosis of NAFLD [3]. With the increasing prevalence of obesity and type 2 diabetes, NAFLD has now become the most common cause of abnormal liver biochemistry (elevated liver transaminases) in western countries [3]. NAFLD ranges from simple fatty liver disease (steatosis), through fat with necro-inflammation and/or fibrosis (non-alcoholic steatohepatisis (NASH)) to advanced fibrosis, cirrhosis and hepato-cellular cancer [5]. In Europe, the prevalence of NAFLD is estimated to be 20–30% in the general population [6]. As it is a reversible condition, early non-invasive detection may play a significant role in the management of NAFLD [7, 8]. NAFLD occurs more frequently in the middle-aged and older populations given that risk factors for its development tend to increase in prevalence with advancing age [9]. Older adults also show more severe biochemical, haematological and histological changes than their younger counterparts[5].

The gold standard technique to detect steatosis is a liver biopsy with histological or biochemical estimation of hepatic fat content [10–12]. However, a liver biopsy is an invasive technique which may be associated with clinical complications [1, 13–15] and thus it is rarely undertaken in the absence of a clear clinical diagnostic imperative. Circulating liver enzyme concentrations such as aspartate transaminase (AST) and alanine transaminase (ALT) are often used as proxy markers of NAFLD[16]. However, these tests have low sensitivity and specificity and may not predict clinical outcomes [17]. Alternatively, imaging methods may be employed to define the extent and course of the disease. The best imaging techniques can be used as reference methods but are not feasible in large-scale population-based studies due to logistical and financial constraints. In addition, Computer tomography (CT) exposes participants to ionising radiation, which limits its use for repeated/longitudinal examinations and research studies [1, 14, 18, 19]. Magnetic resonance imaging (MRI) and proton magnetic resonance spectroscopy (MRS) allow quantification of hepatic fat and are increasingly accepted as non-invasive alternatives to liver biopsy [20, 21], thus permitting longitudinal assessment of hepatic fat in patients. MRS has been shown to be highly reproducible with high diagnostic accuracy [22]

In contrast to the gold standard imaging methods, ultrasonography is a safe, inexpensive and highly accessible imaging technique for the detection of steatosis both in clinical and research settings. Its validity has been tested against those imaging technique in small sample sizes and younger age groups [23–26]; however it is important to assess its validity in older adults due to the anatomical and physiological liver changes with increasing age. Extra-hepatic manifestations and complications, such as cardiovascular disease and extra-hepatic neoplasms are more common in older age groups than their younger counterparts [9].

The reported accuracy and reliability have also been inconsistent across studies as this method is subject to inter-observer variability [7, 27]; and little is known about the performance of the specific ultrasound criteria used to assess hepatic steatosis.

We aimed to assess the diagnostic accuracy and inter-observer reliability of the ultrasound method to detect the presence of steatosis against MRS as the criterion method. In particular, we evaluated whether the ultrasound can predict the grade of severity of steatosis (mild, moderate and severe) and determined the contribution of specific ultrasound criteria to prediction.

## Materials and methods

### Study population

The study was based on data collected on 100 healthy white European individuals, aged 67–76 years, participating in the Hertfordshire Birth Cohort Physical Activity trial [28]. Analyses were performed on the final sample of 72 individuals (n = 42 men; n = 30 women) who had complete data on both MRS and ultrasound. Missing data for MRS scans were due to technical issues or termination of the scan due to participant claustrophobia.

The original exclusion criteria for the trial were inability to cycle unaided for a minimum of 30 minutes; contraindications for physical activity; prevalent diabetes, untreated or unstable ischaemic heart disease, and contraindications to a magnetic resonance scan.

The study was approved by the Hertfordshire Local Research Ethics Committee and was performed in accordance with the Declaration of Helsinki. Written informed consent was obtained from all study participants.

### Study measurements

Volunteers were asked to refrain from eating for 10 hours before their arrival at the clinic as a glucose tolerance test was part of the trial protocol.

#### Anthropometry

All measurements were carried out by trained research staff. Weight was measured on TANITA (model BC-418 MA, Tokyo, Japan) and height with a wall-mounted stadiometer (SECA model 240, Birmingham, UK). Body mass index (BMI) was calculated as weight (kg) divided by height squared (m^2^).

#### Ultrasound

Ultrasound scanning was performed with a LOGIQ Book XP ultrasound device (GE Healthcare) with a 3C-RS curved transducer. A sub-costal approach was used to image both liver lobes using a standard imaging protocol. All liver sweeps were made during deep inspiration. The first sweep was made from lateral to medial with the right lobe in an optimised longitudinal scan plane that showed the liver and long axis of the kidney. The second sweep was made from cranial to caudal, starting at the level of the hepatic veins, along the portal vein towards the gallbladder, with the dome of the liver in a transverse position. The third sweep was a longitudinal scan of the left lobe and was made from lateral to medial just over the gallbladder area. The final sweep was a left transverse scan from the level of the hepatic veins towards the pancreas.

**Fig 1:**
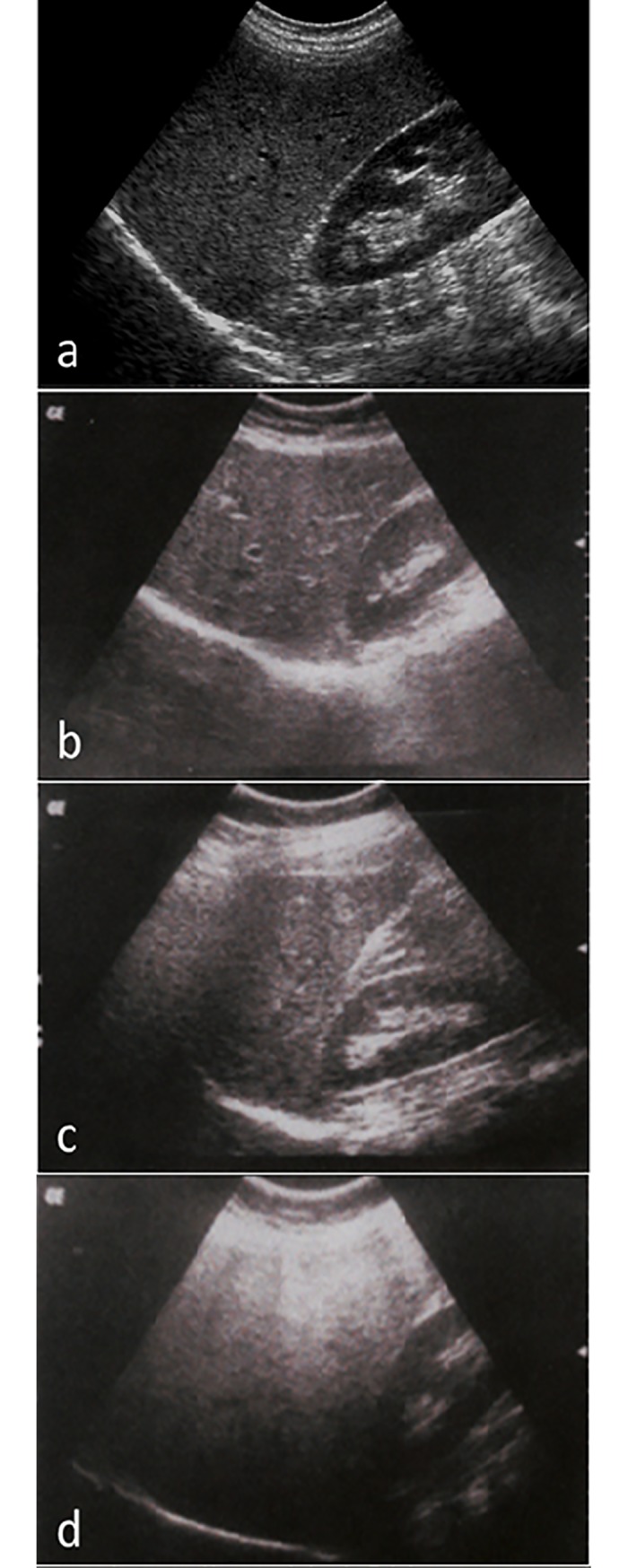
Gradation of hepatic steatosis: a. Normal liver echotexture. Longitudinal section through the right lobe of the liver. Similar echogenicity between liver parenchyma and the cortex of the right kidney. Intra-hepatic vascular anatomy is clearly visible and posterior aspects of the liver are well depicted; b. Mild—slightly increased echogenicity when compared to the renal cortex; blood vessels/diaphragm in view; c. Moderate—clear increased echogenicity of the liver parenchyma, impaired visualisation of intrahepatic vascular anatomy, decreased echogenicity of the renal cortex; d. Severe—marked increased echogenicity/absorption, poor penetration and visualisation of intrahepatic vascular anatomy and diaphragm, marked decreased echogenicity of the renal cortex.

A semi-quantitative grading system was used to define normal echotexture or mild, moderate and severe steatosis (Fig 1).

The images were recorded and scored retrospectively by two operators who were blinded to all other study measures. The ultrasound images were qualitatively scored according to standardised criteria [29–32]. The hepatic steatosis scoring criteria were: a) criterion 1—increased echo reflectivity of the liver parenchyma (bright liver in comparison with the kidney), b) criterion 2—decreased visualisation of the intra-hepatic vasculature, c) criterion 3—attenuation of ultrasound beam. Each criterion was scored on a 4-point scale (i.e. as 1, 2, 3 or 4) and summed, resulting in cumulative liver fat score which ranged from 3 to 12. A score of ≤4 was classified as normal liver, 5–7 as mild steatosis, 8–10 as moderate steatosis, and score ≥11 was classified as severe steatosis. To test whether ultrasound can diagnose the presence or the absence of hepatic steatosis, the mild, moderate and severe categories were grouped together.

The validity of scores of the two operators was assessed by comparison with a third senior radiographer (the gold standard), who had carried out their training. 20 scans were randomly selected from the whole sample and were scored independently by the operators and the reviewer. Correlation coefficients with the reviewer’s scores were 0.97 (p<0.001) and 0.86 (p<0.001) for operator A and B, respectively. The agreement between different readers was substantial; the inter reader agreement between operator A and the gold standard was k = 0.98 between operator B and the gold standard was k = 0.71 and between operator A and B was 0.72.

#### Proton magnetic resonance spectroscopy

Proton MRS measures of intrahepatic lipid (IHL) content were conducted on a Siemens MAGNETOM 3T Tim Trio scanner at the Wolfson Brain Imaging Centre, Cambridge Biomedical Campus, UK. A proton spectrum was obtained from a voxel of cube length 1.5 cm, located within the posterior aspect of the right lobe of the liver, using the point resolved selective spectroscopy (PRESS) sequence. During this scan participants were given breathing instructions with a 7-second cycle, which was designed and gated such that localisation and subsequent data acquisition occurred at the end of expiration. Non-water suppressed data were acquired with repetition time (TR) = 7 s, echo time (TE) = 35 ms and averaged over 64 measures. The voxel was positioned to avoid blood vessels and the biliary tree, using T_2_-weighted HASTE transaxial images that were also acquired in the same phase of respiration. The spectra were analysed in jMRUI version 3.0 [33]and fitted using the AMARES algorithm [34] with prior knowledge. T_2_-correction was applied assuming a T_2_ relaxation time of 27 ms and 61 ms for hepatic water and CH_2_ lipid respectively at 3.0T[35]. IHL was quantified using water as an internal reference [24], and was expressed as the CH_2_ resonance at 1.3 ppm divided by the sum of the CH_2_ and water resonances. Hepatic fat content above 5.5% by MRS was used as a cut-off value for steatosis, which represents the 95^th^ centile of hepatic fat content in low risk individuals with normal BMI, glucose tolerance, liver function tests and low alcohol consumption[36].

## Statistical analysis

Statistical analysis was performed using Stata version 13.0 (StataCorp). Results are presented as means (SD), *n* (%) or median (IQR). The sensitivity and specificity of the ultrasound in predicting the presence of hepatic steatosis was calculated against the MRS normal/abnormal categories. The performance of specific ultrasound criteria was assessed against the content of hepatic fat assessed by MRS.

## Results

Data on 72 individuals (n = 30 women; n = 42 men) were available for analysis, with a mean age of 72±2.5 years and a mean BMI of 26.6 ± 3.8 kg/m^2^. Diabetes was present in 6% of the cohort; none of the participants were underweight or morbidly obese. Mean percentage hepatic steatosis as measured by MRS was 25% (Table 1).

**Table 1:** Characteristics of participants from the Hertfordshire Birth Cohort Physical Activity trial with hepatic steatosis measures by ultrasound and MRS.

Total Number	72
Men	42 (58%)
Age (yrs)	72 ±2.5
BMI (kg/m^2^)	26.6±3.8
Waist (cm)	97.0±12.0
ALT (iu/L)	24 (18–33)
Prevalence of Type 2 Diabetes	4 (6%)
Hepatic steatosis by MRS	18 (25%)
Hepatic steatosis by ultrasound	19 (26%)

Data are mean ± SD, N (%) or median (IQR)

MRS Magnetic Resonance Spectroscopy

ALT Alanine aminotransferase

GGT Gamma-glutamyl transferase

Steatosis was strongly linked to overall and central obesity. It was detected in 64% of the obese participants (BMI ≥30), 26% of the overweight participants (25< BMI <30), and 12% of normal weight participants. Steatosis was also found in 13 men (35%) and in 6 women (24%) with central obesity (waist circumference ≥90 cm in men and ≥80 cm in women). 10% of the study participants who had raised ALT levels had hepatic steatosis (Table 2).

**Table 2:** Prevalence of overweight, obesity, central obesity, and raised ALT levels, overall and by MRS or ultrasound categories of hepatic steatosis.

* *	N	Steatosis by MRS^c^	Steatosis by USS^d^

*Weight status*^*a*^			
Normal weight	26 (36%)	2 (8%)	3 (12%)
Overweight	35 (49%)	10 (29%)	9 (26%)
Obese	11 (15%)	6 (55%)	7 (64%)
*Central obesity*^*b*^			
Central obesity (men)	37 (51%)	13 (35%)	13 (35%)
Central obesity (women)	25 (35%)	5 (20%)	6 (24%)
*ALT* >40 iu/L	7 (10%)	5 (71%)	4 (57%)

^a^Normal weight: BMI 18.5–24.9 kg/m^2^; Overweight: 25.0–29.9 kg/m^2^; Obese: ≥30 kg/m^2^

^b^Central obesity: waist circumference ≥90 cm in men, and ≥80 cm in women

^c^MRS: Magnetic Resonance Spectroscopy

^d^USS: Ultrasound Scan

Abnormal ALT: >40iu/L

**Fig 2:**
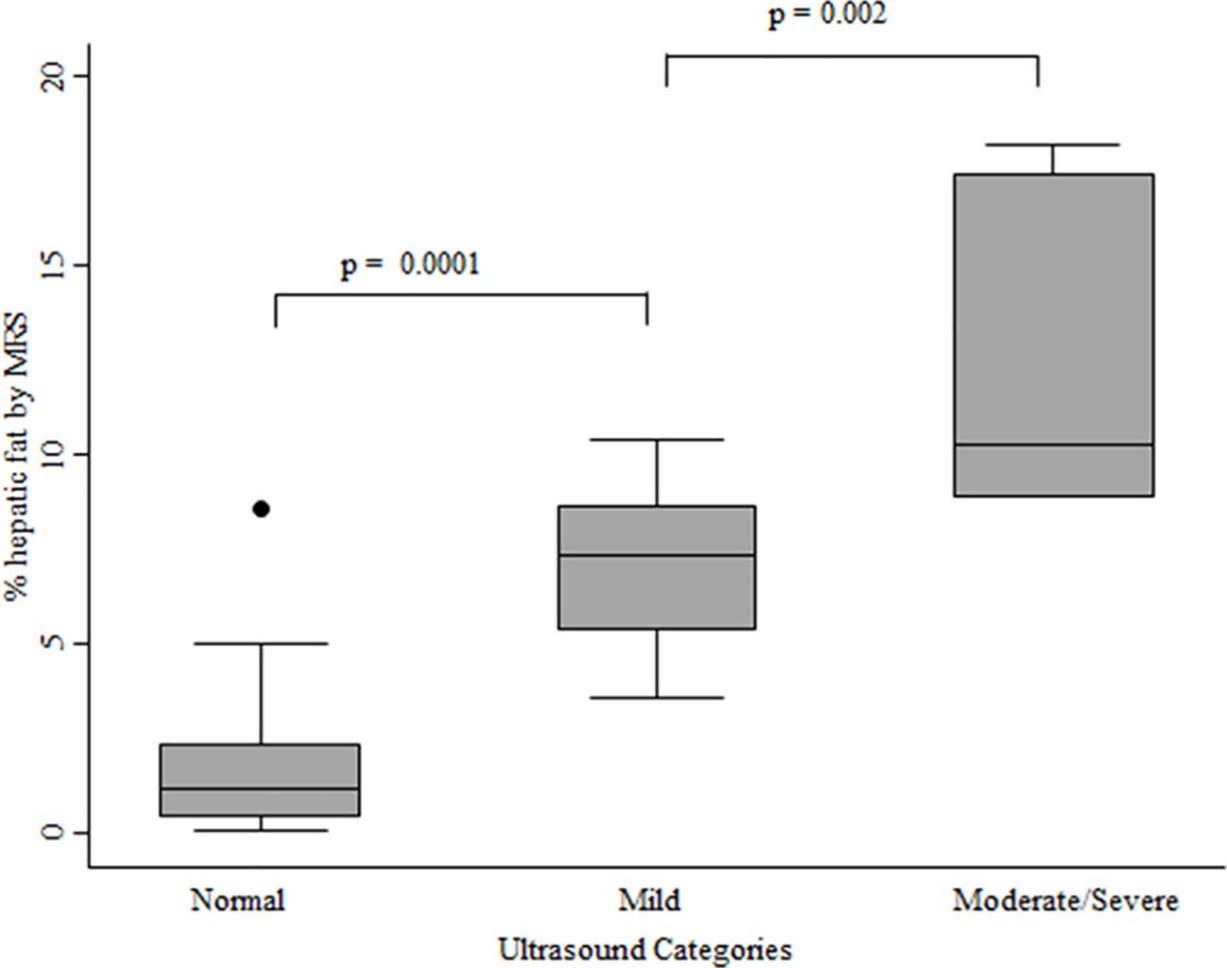
Box and whisker plot of MRS hepatic steatosis levels by ultrasound category. The lower boundary of the box indicates the 25^th^ percentile, the line within the box is the median value and the upper boundary of the box indicates the 75^th^ percentile. The whiskers indicate the minimum and maximum data values. If outliers are present the wiskers extend to the nearest data observation which lies within 1.5 times the interquartile range from the box. The moderate and severe scores were combined due to the small number of individuals in these categories.

Fig 2. illustrates MRS intrahepatic fat levels by ultrasound categories. The moderate and severe categories were grouped together due to small numbers. Median and interquartile ranges (IQR) of MRS hepatic fat content increased progressively from the normal to the mild and moderate/severe categories (normal: 2.2% (1.2 to 3.5%); mild: 8.2% (7.3–15.0%); moderate/severe: 19.5% (18.1–30.4%); p<0.0001).

Table 3 describes the accuracy of the ultrasound method to detect hepatic steatosis versus the criterion method of MRS. The sensitivity and specificity of the ultrasound method in diagnosing presence or absence of any degree of steatosis was 96% (95% CI 87–99.6%) and 94% (95% CI 73–100%) respectively, with a positive predictive value of 98% (95% CI 90–100%).

**Table 3:** Accuracy of ultrasound in detecting hepatic steatosis as defined by MRS in 72 participants of the Hertfordshire Birth Cohort Physical Activity trial.

	MRS categories	
	No steatosis^a^	Steatosis^b^
*Ultrasound categories*:		
Normal	52	1
Mild	2	10
Moderate	0	2
Severe	0	5

^a^No steatosis = ≤ 5.5% intrahepatic fat content assessed by MRS

^b^Steatosis = > 5.5% intrahepatic fat content assessed by MRS

None of the individual ultrasound criteria performed as well as the combined ultrasound assessment (Table 4). For the ultrasound criterion 1 (increased echo reflectivity of the liver parenchyma (bright liver), the sensitivity was higher than the combined score (100%; 95% CI 82–100%), but the specificity was substantially lower (13%; 95% CI 45–25%). The positive predictive value was also lower than the overall score, 28% (95% CI 18–40%). For criterion 2 (decreased visualization of the intra-hepatic vasculature), the sensitivity and specificity for detecting steatosis was 92% (95% CI 82–98%) and 89% (95% CI 65–98) respectively with a positive predictive value of 96% (95% CI 87–100). For criterion 3 (attenuation of the ultrasound beam, impaired penetration) the sensitivity was lower than criterion 1 and 2, 78% (95% CI 52–94), but the specificity was high 98.2% (95% CI 90–100).

**Table 4:** Accuracy of individual ultrasound criterion to detect hepatic steatosis.

	MRS categories	
	No steatosis	Steatosis
*Ultrasound criteria*^*a*^:		
* *		
criterion 1: Echo reflectivity		
Negative	7	0
Positive	47	18
criterion 2: Vasculature		
Negative	50	2
Positive	4	16
criterion 3: Attenuation		
Negative	53	4
Positive	1	14

^a^Ultrasound criteria

criterion 1: Increased echo reflectivity of liver parenchyma (in comparison with the kidney)

criterion 2: Decreased visualisation of intra-hepatic vasculature

criterion 3: Attenuation of ultrasound beam, impaired penetration

## Discussion

Given the on-going obesity epidemic, the prevalence of hepatic steatosis is likely to increase, but accurate data on prevalence are unlikely to be possible in the absence of validated methods that can be applied at the population level. Our study shows that ultrasound is an accurate and reliable imaging method for detecting the presence of hepatic steatosis when compared to MRS, with a sensitivity of 96% and a specificity of 94% and a high positive predictive value (98%). Furthermore, ultrasound allows the assessment of severity as it can discriminate between mild, moderate and severe hepatic steatosis.

Our data are consistent with previous reports which compared ultrasound against histology, MRS or other imaging techniques, suggesting that ultrasound is a valid method to diagnose hepatic steatosis [7, 25–27, 31, 37]. However, other studies have reported that the sensitivity may decrease in obese and older patients [38–40] perhaps because thick layers of subcutaneous fat may limit the ability to reliably detect liver echogenicity[39] or ageing may change the echo properties of the liver and kidney[40]. However, our observations demonstrate that the ultrasound method used here has excellent validity even in a sample of older individuals with a high prevalence of overweight and obesity (overweight 49% and obese 15%).

Our study also found a substantial overlap between the ultrasound-defined categories of steatosis. Similar observations were also reported in another MRS study of 50 adults (43 men, 7 women), in which considerable overlap in hepatic lipid content was observed between different ultrasound grades: absent (0.0–1.58%), mild (2.2–16.2%), moderate(4.9–26.7%) and severe (8.1–76.8%) steatosis [26]. Liver fibrosis and inflammation may affect the ultrasound grades and lead to misclassification; advanced fibrosis could cause echogenic abnormalities on the ultrasound image, decreasing the sensitivity of this method to detect mild to moderate to severe histological steatosis [1]. We also found that the performance of specific individual ultrasound criteria was not as accurate as the combined ultrasound score, a finding similar to a previous report that sensitivity was highest for a combined scoring method [41].

This study had some limitations. We were unable to investigate whether the ultrasound can differentiate between moderate and severe hepatic steatosis as our sample only included 2 individuals in the moderate category and 5 in the severe category. However, a meta-analysis which included 34 studies and 2815 participants [7] found that the overall sensitivity and specificity of the ultrasound in discriminating histologically defined moderate to severe steatosis was 84.8% and 93.6% respectively, suggesting that the ultrasound can be used to classify the degree of severity of hepatic steatosis. Our study sample included older individuals but did not include any participants with severe obesity (BMI≥40), thus we cannot generalise our conclusions about the validity of ultrasound for the assessment of steatosis to that sub-group. This method will need to be validated in independent studies including groups of different ages and body sizes. Further research in this area would be of benefit. Furthermore, this study did not include more objective ultrasound approaches of liver steatosis such as the hepatorenal ratio using histogram echo intensity and transient elastography (Fibroscan or Echosens). Software is typically required to calculate the hepatorenal ratio in large datasets, and this is often not available as freeware. Retrospective assessment of images is not always feasible as technical parameters such as gain and dynamic range require setting at the time of scanning to maximise contrast, making the process of deriving this ratio cumbersome[42]. The applicability of the transient elastography method is questionable in obese individuals (BMI > 30 kg/m^2^) as the fatty thoracic belt attenuates elastic and US waves, making liver stiffness measurement challenging, which may result in underestimating liver damage[42]. In addition, the validation work of this tool has been mainly carried out in patients with chronic liver disease and their results may not be extrapolated to the general population [43–45].

MRS was used as the criterion method in this study as it is non-invasive and it would not have been possible, either ethically or practically, to have used liver biopsy as the criterion method in a study of healthy volunteers from the general population. MRS is an accepted method for accurate detection and quantification of hepatic steatosis [19] and has been validated against biochemical and histological analyses of liver tissue biopsy [14, 36, 46]. A possible advantage of MRS as a criterion method is that the technique may assess a much larger volume of the liver than a biopsy, which could be important if the distribution of liver fat is not uniform [19]. Other strengths of our study include the conduct of the ultrasound examination and the MRS scan on the same day and the reduction of inter-observer variation by the validation of the ultrasound scores of the two operators against an experienced radiographer.

In conclusion, our study in older individuals add to the growing evidence that ultrasound is a valid tool for the assessment of the presence or absence of hepatic steatosis. Compared to other diagnostic methods, ultrasound is a non-invasive, relatively inexpensive, and widely available imaging technique that can be employed in clinical settings and large-scale population-based research studies.
